# 4,6-Dibromo-2,3-dimethyl­phenol

**DOI:** 10.1107/S1600536811003151

**Published:** 2011-02-26

**Authors:** Qiaoru Liu, Jungang Wang, Weijian Xue, Qi Li

**Affiliations:** a School of Chemistry and Chemical Engineering, Pingdingshan University, Pingdingshan 467000, People’s Republic of China; b Key Laboratory of Pesticide and Chemical Biology of the Ministry of Education, College of Chemistry, Central China Normal University, Wuhan 430079, People’s Republic of China

## Abstract

The mol­ecule of the title compound, C_8_H_8_Br_2_O, is approximately planar with a maximum deviation of 0.063 (1) Å for one of the Br atoms. In the crystal, adjacent mol­ecules are joined inter­molecular O—H⋯O hydrogen bonds, forming chains parallel to [010]. The structure also features a short Br⋯Br inter­action of 3.362 (1) Å.

## Related literature

For the synthesis, see: Lai *et al.* (1993 ▶). For a related structure, see: Bringmann & Messer (2001 ▶).
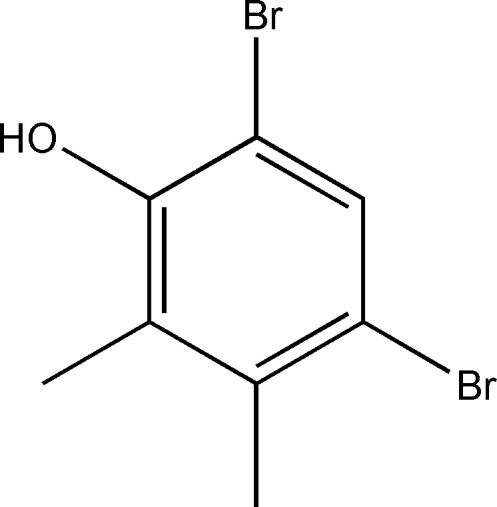


## Experimental

### 

#### Crystal data

C_8_H_8_Br_2_O
*M* *_r_* = 279.96
Monoclinic, 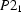

*a* = 7.3604 (5) Å
*b* = 4.4310 (6) Å
*c* = 14.0245 (10) Å
β = 92.482 (1)°
*V* = 456.96 (8) Å^3^
*Z* = 2
Mo *K*α radiation
μ = 8.81 mm^−1^
*T* = 298 K
0.16 × 0.12 × 0.10 mm

#### Data collection

Bruker SMART CCD area-detector diffractometer
Absorption correction: multi-scan (*SADABS*; Sheldrick, 1996 ▶) *T*_min_ = 0.333, *T*_max_ = 0.473
5557 measured reflections
2250 independent reflections
1882 reflections with *I* > 2σ(*I*)
*R*_int_ = 0.042

#### Refinement

*R*[*F* ^2^ > 2σ(*F* ^2^)] = 0.037
*wR*(*F* ^2^) = 0.090
*S* = 0.99
2250 reflections
102 parameters
H-atom parameters constrained
Δρ_max_ = 0.57 e Å^−3^
Δρ_min_ = −0.29 e Å^−3^
Absolute structure: Flack (1983 ▶), 1275 Friedel pairs
Flack parameter: 0.02 (2)

### 

Data collection: *SMART* (Bruker, 1997 ▶); cell refinement: *SAINT* (Bruker, 1999 ▶); data reduction: *SAINT*; program(s) used to solve structure: *SHELXS97* (Sheldrick, 2008 ▶); program(s) used to refine structure: *SHELXL97* (Sheldrick, 2008 ▶); molecular graphics: *SHELXTL* (Sheldrick, 2008 ▶); software used to prepare material for publication: *SHELXTL*.

## Supplementary Material

Crystal structure: contains datablocks I, global. DOI: 10.1107/S1600536811003151/ng5106sup1.cif

Structure factors: contains datablocks I. DOI: 10.1107/S1600536811003151/ng5106Isup2.hkl

Additional supplementary materials:  crystallographic information; 3D view; checkCIF report

## Figures and Tables

**Table 1:** Hydrogen-bond geometry (Å, °)

*D*—H⋯*A*	*D*—H	H⋯*A*	*D*⋯*A*	*D*—H⋯*A*
O1—H1⋯O1^i^	0.82	2.25	2.913 (4)	139

Symmetry code: (i) 
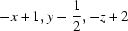
.

**Fig. 1.**
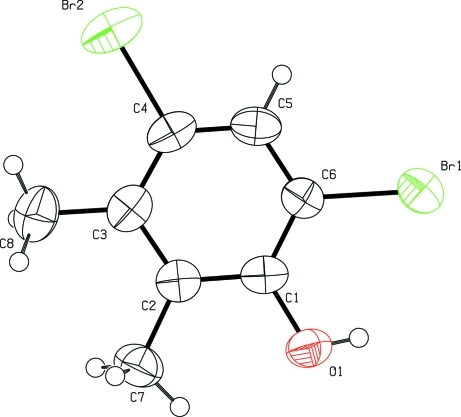
The structure of the title compound, showing 30% probability displacement ellipsoids and the atom-numbering scheme.

**Fig. 2.**
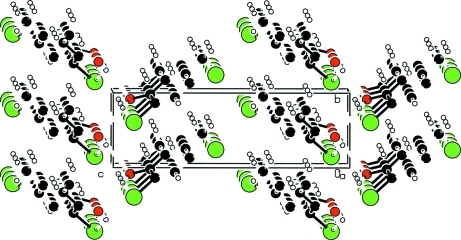
Part of the crystal packing, showing the formation of the one-dimensional chain in (I) by the O1—H1···O1(-x, y - 1/2, 2 - z) hydrogen bond.

## supplementary crystallographic information

### Comment

In the title compound,*C*~8~H~8~Br~2Õ, the adjacent molecules are molecules are joined togethe by the O1—H1···O1 (-*x*, *y* - 1/2, 2 - *z*) hydrogen bond, forming a one-dimensional chain running parallel to the [010] direction(Table 1 and Figure 2). Also Br···Br interaction was observed in (I) with a distance of 3.362 (1) Å between them All the bond lengths and angles are similar to the reported compound (Bringmann *et al.*, 2001).

### Experimental

The title compound, synthesized by 2,3-dimethyl phenylamine through three steps such as bromination, diazotization-bromination-hydrolysis reaction.The operating process was based on the literarure (Lai *et al.*, 1993) and made some improvement.

Firstly, 1-amino-4-bromo-2,3-dimethylbenzene was prepared from 2,3-dimethyl phenylamine as described in the literarure(Lai *et al.*, 1993). Then treatment as follows: Sodiumnitrite (1.75 g, 25 mmol) in water (10 ml) was added dropwise into the rapidly stirring mixture of 40% hydrogen bromide (15 ml) containing l-amino-2,3-dimethylbenzene (5.00 g, 25 mmol). The mixture was kept in an ice-bath stiring for 2 h, while the temperature was kept below 5°C by the addition of pieces of ice. Then added 1.97 g (14 mmol) cuprous bromide which was pretreatmented by refluxing with 10 ml 40% hydrogen bromide solution for 1 h. After the addition the mixture was heated refluxing for an additional 1 h, and then cooled to room temperature, extract by methylenechloride. The organic layer was washed by water, dried by anhydrous natriumsulfate, evaporated under reduced pressure and chromatographed on silica gel with hexane as the eluent. The title compound was obtained as needle crystal solid 1.82 grams. Yield was 26%. Colorless needle-like single crystals suitable for X-ray diffraction studies were obtained by slow evaporation of a solution of the title compound in chloroform: methanol (3: 1) at room temperature.

### Refinement

In (I), all carbon H atoms were positioned geometrically and refined as riding atoms, with C—H = 0.93 Å and *U*\ĩso\~(H) = 1.2*U*\~eq\~(C) for aromatic H atoms, and C—H = 0.96 Å and *U*\ĩso\~(H) =1.5*U*\~eq\~(C) for methyl H atoms. H1 atom was found first from the difference map and placed at its ideal position with the O—H=0.82Å and U\ĩso\~(H)=1.5U\~eq\~(O). The Friedel pairs is 1275.

### Crystal data

**Table:**

C_8_H_8_Br_2_O	*F*(000) = 268
*M**_r_* = 279.96	*D*_x_ = 2.035 Mg m^−^^3^
Monoclinic, *P*2_1_	Mo *K*α radiation, λ = 0.71073 Å
Hall symbol: P 2yb	Cell parameters from 2355 reflections
*a* = 7.3604 (5) Å	θ = 2.8–24.5°
*b* = 4.4310 (6) Å	µ = 8.81 mm^−^^1^
*c* = 14.0245 (10) Å	*T* = 298 K
β = 92.482 (1)°	Needle, colorless
*V* = 456.96 (8) Å^3^	0.16 × 0.12 × 0.10 mm
*Z* = 2	

### Data collection

**Table:**

Bruker SMART CCD area-detector diffractometer	2250 independent reflections
Radiation source: fine-focus sealed tube	1882 reflections with *I* > 2σ(*I*)
graphite	*R*_int_ = 0.042
phi and ω scans	θ_max_ = 28.3°, θ_min_ = 2.8°
Absorption correction: multi-scan (*SADABS*; Sheldrick, 1996)	*h* = −9→9
*T*_min_ = 0.333, *T*_max_ = 0.473	*k* = −5→5
5557 measured reflections	*l* = −18→18

### Refinement

**Table:**

Refinement on *F*^2^	Secondary atom site location: difference Fourier map
Least-squares matrix: full	Hydrogen site location: inferred from neighbouring sites
*R*[*F*^2^ > 2σ(*F*^2^)] = 0.037	H-atom parameters constrained
*wR*(*F*^2^) = 0.090	*w* = 1/[σ^2^(*F*_o_^2^) + (0.0403*P*)^2^] where *P* = (*F*_o_^2^ + 2*F*_c_^2^)/3
*S* = 0.99	(Δ/σ)_max_ = 0.001
2250 reflections	Δρ_max_ = 0.57 e Å^−^^3^
102 parameters	Δρ_min_ = −0.29 e Å^−^^3^
0 restraints	Absolute structure: Flack (1983), 1275 Friedel pairs
Primary atom site location: structure-invariant direct methods	Flack parameter: 0.02 (2)

### Special details

**Table:**

Geometry. All e.s.d.'s (except the e.s.d. in the dihedral angle between two l.s. planes) are estimated using the full covariance matrix. The cell e.s.d.'s are taken into account individually in the estimation of e.s.d.'s in distances, angles and torsion angles; correlations between e.s.d.'s in cell parameters are only used when they are defined by crystal symmetry. An approximate (isotropic) treatment of cell e.s.d.'s is used for estimating e.s.d.'s involving l.s. planes.
Refinement. Refinement of *F*^2^ against ALL reflections. The weighted *R*-factor *wR* and goodness of fit *S* are based on *F*^2^, conventional *R*-factors *R* are based on *F*, with *F* set to zero for negative *F*^2^. The threshold expression of *F*^2^ > σ(*F*^2^) is used only for calculating *R*-factors(gt) *etc*. and is not relevant to the choice of reflections for refinement. *R*-factors based on *F*^2^ are statistically about twice as large as those based on *F*, and *R*- factors based on ALL data will be even larger.

### Fractional atomic coordinates and isotropic or equivalent isotropic displacement parameters (Å^2^)

**Table:**

	*x*	*y*	*z*	*U*_iso_*/*U*_eq_	
Br1	0.87217 (5)	−0.34219 (12)	0.92038 (3)	0.05521 (15)	
Br2	0.86105 (8)	0.29262 (15)	0.57447 (4)	0.0790 (2)	
C1	0.5729 (5)	0.0139 (10)	0.8491 (3)	0.0436 (9)	
C2	0.4789 (5)	0.2000 (10)	0.7846 (3)	0.0458 (10)	
C3	0.5600 (6)	0.2866 (12)	0.6995 (3)	0.0496 (9)	
C4	0.7350 (6)	0.1753 (13)	0.6843 (3)	0.0516 (9)	
C5	0.8255 (5)	−0.0114 (11)	0.7471 (3)	0.0498 (10)	
H5	0.9405	−0.0835	0.7342	0.060*	
C6	0.7440 (5)	−0.0922 (9)	0.8300 (3)	0.0429 (9)	
C7	0.2920 (6)	0.3103 (13)	0.8071 (4)	0.0621 (12)	
H7A	0.2479	0.1971	0.8597	0.093*	
H7B	0.2110	0.2837	0.7522	0.093*	
H7C	0.2978	0.5204	0.8237	0.093*	
C8	0.4589 (8)	0.4836 (14)	0.6281 (4)	0.0684 (14)	
H8A	0.5442	0.5917	0.5911	0.103*	
H8B	0.3849	0.6245	0.6610	0.103*	
H8C	0.3828	0.3605	0.5866	0.103*	
O1	0.4886 (4)	−0.0551 (8)	0.9326 (2)	0.0533 (8)	
H1	0.5505	−0.1777	0.9634	0.080*	

### Atomic displacement parameters (Å^2^)

**Table:**

	*U*^11^	*U*^22^	*U*^33^	*U*^12^	*U*^13^	*U*^23^
Br1	0.0508 (2)	0.0528 (2)	0.0620 (3)	0.0041 (2)	0.00077 (17)	−0.0045 (2)
Br2	0.0927 (4)	0.0918 (4)	0.0547 (3)	−0.0172 (3)	0.0285 (3)	−0.0037 (3)
C1	0.043 (2)	0.0419 (19)	0.046 (2)	−0.0093 (17)	0.0072 (16)	−0.0128 (19)
C2	0.047 (2)	0.042 (3)	0.049 (2)	−0.0074 (17)	0.0016 (16)	−0.0067 (17)
C3	0.061 (2)	0.044 (2)	0.043 (2)	−0.012 (2)	−0.0015 (18)	−0.007 (2)
C4	0.059 (2)	0.054 (2)	0.043 (2)	−0.015 (2)	0.0118 (16)	−0.004 (2)
C5	0.045 (2)	0.049 (2)	0.056 (3)	−0.006 (2)	0.0103 (18)	−0.014 (2)
C6	0.043 (2)	0.042 (2)	0.043 (2)	−0.0011 (17)	−0.0010 (16)	−0.0099 (18)
C7	0.046 (2)	0.066 (3)	0.074 (3)	0.004 (2)	0.007 (2)	0.001 (3)
C8	0.089 (4)	0.063 (3)	0.052 (3)	−0.001 (3)	−0.006 (3)	0.002 (3)
O1	0.0545 (17)	0.0582 (19)	0.0482 (18)	0.0023 (15)	0.0143 (13)	0.0007 (15)

### Geometric parameters (Å, °)

**Table:**

Br1—C6	1.903 (4)	C5—C6	1.379 (6)
Br2—C4	1.905 (4)	C5—H5	0.9300
C1—C6	1.381 (5)	C7—H7A	0.9600
C1—O1	1.383 (5)	C7—H7B	0.9600
C1—C2	1.387 (6)	C7—H7C	0.9600
C2—C3	1.411 (6)	C8—H8A	0.9600
C2—C7	1.507 (6)	C8—H8B	0.9600
C3—C4	1.404 (7)	C8—H8C	0.9600
C3—C8	1.501 (7)	O1—H1	0.8200
C4—C5	1.361 (7)		
			
C6—C1—O1	122.4 (4)	C5—C6—Br1	119.5 (3)
C6—C1—C2	120.6 (4)	C1—C6—Br1	119.9 (3)
O1—C1—C2	117.1 (3)	C2—C7—H7A	109.5
C1—C2—C3	119.8 (4)	C2—C7—H7B	109.5
C1—C2—C7	119.4 (4)	H7A—C7—H7B	109.5
C3—C2—C7	120.9 (4)	C2—C7—H7C	109.5
C4—C3—C2	117.2 (4)	H7A—C7—H7C	109.5
C4—C3—C8	122.4 (4)	H7B—C7—H7C	109.5
C2—C3—C8	120.4 (4)	C3—C8—H8A	109.5
C5—C4—C3	122.8 (4)	C3—C8—H8B	109.5
C5—C4—Br2	116.6 (3)	H8A—C8—H8B	109.5
C3—C4—Br2	120.6 (4)	C3—C8—H8C	109.5
C4—C5—C6	119.0 (4)	H8A—C8—H8C	109.5
C4—C5—H5	120.5	H8B—C8—H8C	109.5
C6—C5—H5	120.5	C1—O1—H1	109.5
C5—C6—C1	120.6 (4)		
			
C6—C1—C2—C3	−1.2 (6)	C2—C3—C4—Br2	−177.2 (3)
O1—C1—C2—C3	178.0 (4)	C8—C3—C4—Br2	4.2 (7)
C6—C1—C2—C7	179.6 (4)	C3—C4—C5—C6	−1.1 (7)
O1—C1—C2—C7	−1.3 (6)	Br2—C4—C5—C6	177.0 (3)
C1—C2—C3—C4	0.3 (6)	C4—C5—C6—C1	0.2 (6)
C7—C2—C3—C4	179.6 (4)	C4—C5—C6—Br1	−177.9 (3)
C1—C2—C3—C8	178.9 (4)	O1—C1—C6—C5	−178.2 (4)
C7—C2—C3—C8	−1.8 (7)	C2—C1—C6—C5	0.9 (6)
C2—C3—C4—C5	0.8 (7)	O1—C1—C6—Br1	−0.1 (5)
C8—C3—C4—C5	−177.8 (5)	C2—C1—C6—Br1	179.0 (3)

### Hydrogen-bond geometry (Å, °)

**Table:**

*D*—H···*A*	*D*—H	H···*A*	*D*···*A*	*D*—H···*A*
O1—H1···O1^i^	0.82	2.25	2.913 (4)	139.
O1—H1···Br1	0.82	2.57	3.108 (3)	124.
C8—H8A···Br2	0.96	2.70	3.200 (6)	113.

Symmetry codes: (i) −*x*+1, *y*−1/2, −*z*+2.
